# Paradoxical association between campus connectedness and delusion-like experiences among Chinese college students: a chained mediation

**DOI:** 10.3389/fpsyg.2026.1769942

**Published:** 2026-05-08

**Authors:** Jiawei Xing, Juan Li

**Affiliations:** 1 Educational Faculty, Jiangxi Science and Technology Normal University, Nanchang, China; 2 School of Health Care Security, Shandong First Medical University, Jinan, China

**Keywords:** campus connectedness, chained mediation, delusion-like experiences, depression, insomnia

## Abstract

**Introduction:**

Psychotic-like experiences are common among college students, and the delusion-like dimension is an important component of this spectrum. However, little is known about how campus-related protective factors are associated with delusion-like experiences in this population. Campus connectedness, reflecting students’ connection and integration within the campus community, may be negatively associated with delusion-like experiences. Insomnia and depression, both highly relevant to college student mental health, may help account for this association, not only as individual mediators but also as part of a sequential pathway. This study therefore examined the association between campus connectedness and delusion-like experiences, with particular attention to the hypothesized chained mediation involving insomnia and depression.

**Methods:**

The sample of this study consisted of 1,048 college students (Mage = 19.6 years, SD = 1.75). Data on variables were collected through an online questionnaire, namely campus connectedness, insomnia, depression, delusion-like experiences, and other demographic variables. Structural equation modeling was used for data analysis, and relevant statistical analyses were conducted with SPSS 25.0 and Mplus 8.3.

**Results:**

Campus connectedness was negatively correlated with delusion-like experiences, whereas insomnia and depression were positively correlated with delusion-like experiences. The findings were consistent with significant indirect associations through insomnia, depression, and their hypothesized sequence. After both mediators were included, the residual direct association between campus connectedness and delusion-like experiences changed sign from negative to positive, suggesting a suppression-like pattern.

**Conclusion:**

The findings suggest that the association between campus connectedness and delusion-like experiences may partly operate through insomnia and depression, particularly through their hypothesized chained mediation, and may be more complex than a uniformly protective model would imply. These results should be interpreted cautiously, given the cross-sectional design.

## 1 Introduction

Psychotic-like experiences (PLEs), which refer to subclinical manifestations of psychotic symptoms, have been documented in college populations (Fonseca-Pedrero et al., 2016; Fekih-Romdhane et al., 2021; Korenic et al., 2021; Sun et al., 2021; Xu et al., 2024). Previous studies have shown that persistent PLEs are associated with an elevated risk of subsequent psychotic disorders and other serious mental health problems (van Os et al., 2009; Yung et al., 2009). Within the PLE spectrum, delusion-like experiences constitute an important dimension (van Os and Kapur, 2009; Zamperoni et al., 2022; Granö et al., 2024). Delusion-like experiences refer to subclinical forms of delusional ideation, including ideas of reference, persecutory ideas, or beliefs of being controlled, that do not necessarily meet diagnostic thresholds (Freeman, 2007; Miyazono and Salice, 2021; Bansal et al., 2022; Solomonova et al., 2025). Current research indicates that delusion-like experiences are better characterized as modest, heterogeneous, and domain-specific rather than as reflecting generalized cognitive impairment. Cognition-related correlates appear more likely to involve specific executive and reasoning-related processes, and many individuals with subclinical psychotic experiences show broadly intact cognitive functioning (Mollon et al., 2016; Chu et al., 2025).

Recent evidence further suggests that these experiences affect a nontrivial proportion of college students. In a large sample of Chinese college students, 39.5% reported at least one delusion-like experience in the past month and 7.1% reported frequent delusion-like experiences (Xu et al., 2024). Using a broader endorsement criterion, another study found that 59.2% of college students endorsed at least one delusion-like experience item, whereas 13.9% reported frequent delusion-like experiences (Lu et al., 2023). Although these estimates vary across studies depending on the time frame and threshold used, they collectively indicate that delusion-like experiences are sufficiently common in college populations to warrant focused investigation.

Delusion-like experiences are shaped not only by intrapersonal characteristics but also by the broader social environments in which individuals are embedded. Prior research suggests that adverse social experiences, particularly those involving low support, weak belongingness, or interpersonal stress, have been linked to delusion-like experiences, potentially through maladaptive interpretations of social information (Irwin et al., 2024). For college students, the campus is one of the most salient social environments in daily life, serving as a major context for social interaction, belonging formation, and institutional integration (Kassab et al., 2024). This may be particularly true in China, where many universities and colleges operate under a semi-closed campus management system (Ou et al., 2025). In this context, campus connectedness is particularly relevant. It refers to students’ sense of belonging to their institution, perceived fit with others on campus, and social embeddedness in campus life (Lee and Davis, 2000; Jouriles et al., 2020; Johnson and Plisco, 2025). Although a sense of belonging to campus is an important dimension of campus connectedness, the concept extends beyond belonging alone to include students’ supportive relational ties and broader social embeddedness within the campus community (Thelamour et al., 2019; Jouriles et al., 2020; Gao et al., 2022). As a campus-based contextual resource, campus connectedness may contribute to college students’ mental health by fostering interpersonal integration, supporting adjustment to campus life, and strengthening psychological wellbeing (Adams et al., 2021; Costello et al., 2022; Di Malta et al., 2022; Johnson and Plisco, 2025). Accordingly, campus connectedness may serve as an important contextual protective factor against delusion-like experiences among college students.

As a contextual resource, campus connectedness may be associated with lower levels of delusion-like experiences not only directly but also indirectly through more proximal risk factors. In this regard, insomnia and depression may be especially relevant, as previous research has shown that lower campus connectedness might be associated with poorer sleep (Hsieh et al., 2019; Bao et al., 2022; Sun et al., 2025) and more severe depressive symptoms (Gaweda et al., 2013; Wilson et al., 2018; Raymond-Flesch et al., 2021), while both insomnia (Goeder et al., 2021; Kammerer et al., 2021; Sheffield et al., 2021) and depression (Khoury et al., 2023; Koposov et al., 2025) are closely related to delusion-like experiences. Moreover, insomnia and depression are highly prevalent among college students, underscoring their relevance in this population (Jiang et al., 2015; Li et al., 2022). Reports from multiple countries have consistently shown that sleep problems and depressive symptoms are highly prevalent in college populations, underscoring the practical relevance of examining these variables in this context (American College Health Association, 2022, 2023; Sun and Jiang, 2025; Terveyden Ja Hyvinvoinnin Laitos, 2025). In addition, insomnia is often linked to subsequent depressive symptoms (Chen et al., 2021; Hong et al., 2021; Jernelov et al., 2021). Therefore, insomnia and depression may not only function as independent mediators in the association between campus connectedness and delusion-like experiences, but may also jointly form a chained mediation pathway.

**Figure 1:**
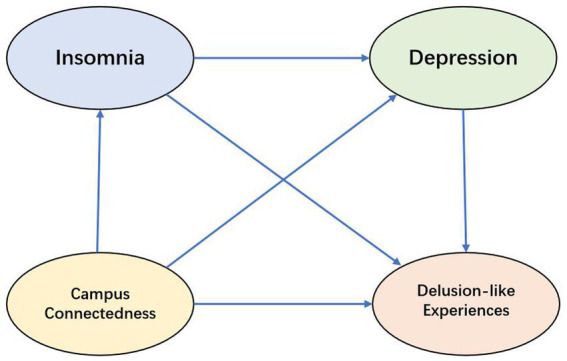
Hypothetical model.

Accordingly, the present study proposes the following hypotheses, as shown in Figure 1.

*H1*: Campus connectedness exerts a negative influence on delusion-like experiences among college students.

*H2*: Insomnia mediates the relationship between campus connectedness and delusion-like experiences among college students.

*H3*: Depression mediates the relationship between campus connectedness and delusion-like experiences among college students.

*H4*: Insomnia and depression exert a chain-mediating effect on the relationship between campus connectedness and delusion-like experiences among college students.

## 2 Methods

### 2.1 Participants and procedure

Participants were recruited from higher education institutions in China using a combination of convenience sampling and snowball dissemination. The research team contacted university administrators, counselors, homeroom teachers, and course instructors across institutions and invited them to distribute the study announcement and survey link through routine teaching and student management channels, such as class group messages and course notifications. Data were collected from February to March 2022. Participants completed a self-administered web-based questionnaire, which required approximately 6 min to complete. The announcement explained the purpose of the study, emphasized that participation was voluntary, stated that responses would be anonymous and confidential, and informed students that they could withdraw at any time without academic or administrative penalty. A total of 1,048 valid responses were obtained.

Table 1 presents the self-reported sociodemographic and socioeconomic characteristics of the sample. Overall, the sample was predominantly female (67.5%) and relatively young, with participants ranging in age from 17 to 26 years (M_age = 19.6, SD = 1.75). First-year students constituted the largest subgroup (54.0%), and most participants were enrolled in undergraduate institutions (71.0%). Monthly household income was classified into five ordinal categories and used as a rough indicator of socioeconomic background. In this sample, 79.1% of participants reported a monthly household income of ¥6,000 or below, indicating that the sample was concentrated among households with relatively limited economic resources in China. Additionally, participants from the provinces of Fujian, Hainan, Henan, and Guizhou accounted for 91.9% of the sample. This study was reviewed and approved by the Academic Ethics Committee of Shandong First Medical University (Approval No. R202202220014).

**Table 1:** Sociodemographic and socioeconomic characteristics of the sample.

Variables	Variable labels	Frequency	Percentage (%)
Gender	Male	341	32.5
	Female	707	67.5
	Total	1,048	100
Age	17	68	6.5
	18	214	20.4
	19	285	27.2
	20	202	19.3
	21	142	13.5
	22 and above	137	13.1
	Total	1,048	100
Grade	1	566	54.0
	2	201	19.2
	3	218	20.8
	4	63	6.0
	Total	1,048	100
HEIC	Undergraduate institutions	744	71.0
	Junior colleges	304	29.0
	Total	1,048	100
MHI	¥ 0–2,000	305	29.1
	¥ 2,001–4,000	339	32.3
	¥ 4,001–6,000	186	17.7
	¥ 6,001–8,000	114	10.9
	¥ 8,001 and above	104	9.9
	Total	1,048	100
Province	Fujian	204	19.5
	Guizhou	312	29.8
	Hainan	313	29.9
	Henan	133	12.6
	Other provinces	86	8.2
	Total	1,048	100

HEIC, higher education institution category; MHI, monthly household income. Monthly household income was grouped into broad ordinal categories to provide a general socioeconomic profile and should not be interpreted as a precise economic indicator.

### 2.2 Instruments

The questionnaire was divided into two parts. Part 1 requested participants’ basic information, including sex, age, grade, institution category, and monthly household income. Part 2 assessed four constructs: campus connectedness, insomnia, depressive symptoms, and delusion-like experiences. In the present web-based survey, all items from these four measures were administered using a harmonized 7-point response format. Specifically, participants were asked to indicate the extent to which each statement described their actual experience or situation. Response options ranged from 1 = did not describe my experience at all to 7 = described my experience completely. We adopted this unified format to maintain response consistency across measures within the same questionnaire and to provide sufficient response discrimination.

The Campus Connectedness Scale (CCS) was developed by Yu et al. based on prior research and has demonstrated good applicability among Chinese college students (Gao et al., 2022). This scale comprises three factors, namely teacher support (e.g., “Do your teachers treat students fairly?”), peer support (e.g., “Is there someone at school who is willing to help you when you need assistance?”), and sense of belonging (e.g., “Do you feel happy at school?”). Higher scores indicate stronger campus connectedness. The Cronbach’s alpha for the CCS was 0.882, demonstrating good model fit (RMSEA = 0.058, CFI = 0.966, TLI = 0.949, SRMR = 0.034), meeting the requirements for confirmatory factor analysis.

The Youth Self-Report Insomnia Scale (YSIS) was used to measure insomnia among college students. Based on prior research, the items of the YSIS represent two factors—daytime anxiety and insomnia symptoms (Liu et al., 2019). Higher scores indicate more severe insomnia. The Cronbach’s alpha for the YSIS was 0.875, with satisfactory model fit (RMSEA = 0.053, CFI = 0.986, TLI = 0.977, SRMR = 0.026), meeting the requirements for confirmatory factor analysis.

The items developed by Dhir et al. were used to measure students’ depressive symptoms (Dhir et al., 2018). Higher scores indicate more severe depression. Cronbach’s alpha was 0.834, and the model fit was good (RMSEA = 0.038, CFI = 0.995, TLI = 0.990, SRMR = 0.013), meeting the requirements for confirmatory factor analysis.

Sun et al.’s items were incorporated to measure the severity of delusion-like experiences (Sun et al., 2017), which was relatively suitable for Chinese adolescents and college students (Sun et al., 2015). Based on prior research, delusion-like experiences comprise two factors, namely bizarre (thought withdrawal, insertion, and broadcasting) and non-bizarre (thought referencing and control) (Schreier et al., 2009). Higher scores indicate more severe delusional symptoms. Cronbach’s alpha was 0.770. The measure showed acceptable overall model fit (RMSEA = 0.079, CFI = 0.958, TLI = 0.921, SRMR = 0.036).

### 2.3 Statistical analyses

Data were analyzed using structural equation modeling (SEM). Descriptive statistics for campus connectedness, insomnia, depression, delusion-like experiences, and monthly household income were computed in SPSS Version 25, including means, standard deviations, skewness, and kurtosis. Mplus 8.3 was used to estimate bivariate Pearson correlations, assess common method variance (CMV) via a unmeasured latent method construct (ULMC) analysis, and fit the measurement and structural models.

In the SEM analyses, campus connectedness, insomnia, depression, and delusion-like experiences were specified as latent variables. All items were administered using a harmonized 7-point response format and were treated as approximately continuous. For the measurement model, we used theory-driven, dimension-based parceling only for scales with established subdimensions. Specifically, this approach was used only for constructs with theoretically established subdimensions: campus connectedness was represented by three dimension-based indicators, and insomnia and delusion-like experiences were each represented by two dimension-based indicators, whereas depression was represented by its original items. This approach was adopted to preserve the conceptual structure of the scales and to model these constructs at the subdimension level rather than for item-level psychometric revalidation. Accordingly, the findings for these constructs should be interpreted primarily at the subdimension level.

To test the measurement model and the chain-mediated structural model, we used the robust maximum likelihood estimator (MLM), which yields maximum likelihood parameter estimates with robust standard errors and a mean-adjusted chi-square test statistic under mild non-normality. Model fit was evaluated using χ^2^/df, CFI, TLI, RMSEA, and SRMR. For the measurement models, standardized and unstandardized factor loadings, along with their standard errors and significance levels, were examined. To further examine discriminant validity, the hypothesized four-factor measurement model was compared with a series of alternative three-factor, two-factor, and one-factor models. The same fit indices (χ^2^/df, CFI, TLI, RMSEA, and SRMR) were used to evaluate and compare these competing models. No additional demographic covariates (e.g., sex, age, grade, or higher education institution category) were included in the primary SEM, because the model was specified *a priori* to examine the hypothesized relations among the focal constructs. This decision was theory-driven, as methodological research has cautioned against the automatic inclusion of control variables without clear conceptual justification, given the risk of overcontrol, uninterpretable parameter estimates, and misleading inferences (Spector and Brannick, 2011; Becker et al., 2016; Li, 2021).

## 3 Results

### 3.1 CMV test

Common method bias was further assessed using an item-level ULMC analysis. Compared with the baseline trait-only CFA model, the model including a common method factor showed only modest fit improvement (CFI: 0.928–0.954; TLI: 0.918–0.942; RMSEA: 0.055–0.047; SRMR: 0.047–0.032). These results suggest that common method bias, although not fully excludable, was unlikely to substantially account for the main findings.

### 3.2 Symptom characteristics of the sample

Table 2 presents the symptom characteristics of the sample. Overall, participants showed predominantly low-to-moderate levels of insomnia, depression, and delusion-like experiences. Median scores were 3.13 for insomnia, 2.60 for depression, and 3.00 for delusion-like experiences, indicating that the central tendency of symptom endorsement was generally below the higher end of the scale. Consistently, the proportion of participants in the high category was relatively limited across the three core constructs, ranging from 4.4% to 9.4%. A similar pattern was observed for the subdimensions, suggesting that although these symptoms were not absent in the sample, severe endorsement was concentrated in a relatively small subgroup.

**Table 2:** Symptom characteristics of the sample.

Construct	Mean (SD)	Median (Q1, Q3)	Min–max	Low, n(%)	Moderate, n(%)	High, n(%)
Insomnia	3.21 (1.88)	3.13 [2.00,4.00]	1–7	470, 44.8%	480, 45.8%	98, 9.4%
DA	3.43 (1.89)	3.40 [2.20,4.40]	1–7	394, 37.6%	474, 45.2%	180, 17.2%
IS	2.61 (1.74)	2.33 [1.33,3.67]	1–7	620, 59.2%	347, 33.1%	81, 7.7%
Depression	2.73 (1.72)	2.60 [1.60,3.80]	1–7	597, 57.0%	387, 36.9%	64, 6.1%
DLEs	2.83 (1.74)	3.00 [2.17,3.83]	1–7	492, 46.9%	510, 48.7%	46, 4.4%
B	3.33 (1.75)	3.33 [2.67,4.00]	1–7	349, 33.3%	596, 56.9%	103, 9.8%
NB	2.71 (1.68)	2.67 [1.67,3.67]	1–7	581, 55.4%	412, 39.3%	55, 5.2%

DA, daytime anxiety; IS, insomnia symptoms; DLEs, delusion-like experiences; B, bizarre delusion-like experiences; NB, non-bizarre delusion-like experiences. Low, moderate, and high categories were created for descriptive purposes based on mean item scores, with low defined as 1.00–2.99, moderate as 3.00–4.99, and high as 5.00–7.00.

### 3.3 Descriptive statistics

Table 3 presents the mean, standard deviation, skewness, and kurtosis for campus connectedness, insomnia, depression, and delusion-like experiences, along with their Pearson correlation coefficients. Means ranged from 2.73 to 5.27, and standard deviations ranged from 1.39 to 1.88. Skewness and kurtosis values ranged from −0.86 to 0.75 and −1.11 to 0.56, respectively. The Pearson correlation coefficients ranged from −0.713 to 0.820. There was a significant positive correlation among insomnia, depression and delusion-like experiences. In contrast, they all showed a significant negative correlation with campus connectedness.

**Table 3:** Descriptive statistics and Pearson correlation.

CON	M	SD	SKEW	KURT	1	2	3	4	5
1. CC	5.27	1.39	−0.86	0.56	1				
2. INS	3.21	1.88	0.35	−1.11	−0.442^***^	1			
3. DEP	2.73	1.72	0.75	−0.47	−0.713^***^	0.651^***^	1		
4. DLEs	2.83	1.74	0.55	−0.82	−0.470^***^	0.790^***^	0.820^***^	1	

CON, construct; M, mean; SD, standard deviation; SKEW, skewness; KURT, kurtosis; CC, campus connectedness; INS, insomnia; DEP, depression; DLEs, delusion-like experiences. ***p < 0.001, **p < 0.05.

### 3.4 Model fit test

Since the skewness and kurtosis values suggested only mild non-normality, the chain mediation SEM was estimated using the robust maximum likelihood estimator (MLM). The SEM fit was assessed using the following indices: χ2/df = 3.71, RMSEA = 0.051, CFI = 0.977, TLI = 0.966, and SRMR = 0.027. All fit indices met the recommended thresholds.

### 3.5 Measurement model analysis

Based on previous studies, the measurement model analysis divided the items for campus connectedness, insomnia, and delusion-like experiences into different dimensions as indicators. The item parceling method was used to obtain these indicators. According to the existing literature, campus connectedness was divided into peer support, teacher support, and sense of belonging. Insomnia was divided into daytime anxiety and nighttime insomnia. Delusion-like experiences were categorized into bizarre and non-bizarre types. Depression symptoms were not subdivided. Table 4 presents the unstandardized estimates for each measurement model indicator, ranging from 0.83 to 1.09 and demonstrating statistical significance (*p* < 0.001). Standardized estimates fell between 0.65 and 0.86, meeting SEM requirements.

**Table 4:** Measurement model indicators.

Item	Std.	Unstd.	SE	Unstd./SE	*p*
PS	0.82	1.00	0.000	999.00	999.000
SB	0.85	1.09	0.041	26.47	<0.001
TS	0.65	0.89	0.044	20.08	<0.001
DA	0.86	1.00	0.000	999.00	999.000
NI	0.79	0.91	0.036	25.339	<0.001
DEP1	0.72	1.00	0.000	999.000	999.000
DEP2	0.79	1.02	0.040	25.665	<0.001
DEP3	0.72	0.88	0.039	22.855	<0.001
DEP4	0.77	0.90	0.039	22.989	<0.001
BD	0.84	1.00	0.000	999.000	999.000
NBD	0.75	0.83	0.031	26.451	<0.001

Std., standardized estimate; Unstd., unstandardized estimate; SE, standard error; Unstd./SE, ratio of unstandardized estimate to standard error; PS, peer support; SB, sense of belonging; TS, teacher support; DA, daytime anxiety; NI, nighttime insomnia; DEP1–DEP4, depression Indicators; BD, bizarre delusion-like experiences; NBD, non-bizarre delusion-like experiences.

### 3.6 Competing measurement models

To further examine discriminant validity, the hypothesized 4-factor measurement model was compared with a series of alternative 3-factor, 2-factor, and 1-factor models. As shown in Table 5, the hypothesized 4-factor model demonstrated the best overall fit to the data and was the only model that satisfied all prespecified fit criteria. In contrast, all converged alternative models showed poorer fit. These findings provide support for the empirical distinctiveness of the four focal constructs. Additionally, as an auxiliary check of multicollinearity, inner VIF values were examined in SmartPLS 4.1.0.8 for the predictor constructs in the structural model. All VIF values were low (1.000–1.939), suggesting that problematic collinearity was unlikely to have materially distorted the structural path estimates (Supplementary Table S1).

**Table 5:** Fit indices for the competing measurement models.

Model	Description	χ^2^/df	RMSEA	CFI	TLI	SRMR
4-factor model	Hypothesized model	3.72	0.051	0.977	0.966	0.027
3-factor model A	CC + INS combined	18.5	0.129	0.839	0.784	0.096
3-factor model B	CC + DEP combined	Did not converge				
3-factor model C	CC + DELs combined	17.51	0.125	0.848	0.796	0.076
3-factor model D	INS + DEP combined	10.87	0.097	0.909	0.878	0.054
3-factor model E	INS + DELs combined	6.76	0.074	0.947	0.929	0.037
3-factor model F	DEP + DELs combined	8.18	0.083	0.934	0.911	0.049
2-factor model A	CC + INS + DEP combined	Did not converge				
2-factor model B	CC + INS + DELs combined	Did not converge				
2-factor model C	CC + DEP + DELs combined	Did not converge				
2-factor model D	INS + DEP + DELs combined	13.22	0.105	0.888	0.856	0.060
1-factor model	All combined	22.29	0.143	0.789	0.737	0.085

CC, campus connectedness; INS, insomnia; DEP, depression; DLEs, delusion-like experiences. In the competing models, constructs connected by a plus sign were specified as one latent factor. RMSEA, root mean square error of approximation; CFI, comparative fit index; TLI, Tucker-Lewis index; SRMR, standardized root mean square residual.

### 3.7 Structural model analysis

**Figure 2:**
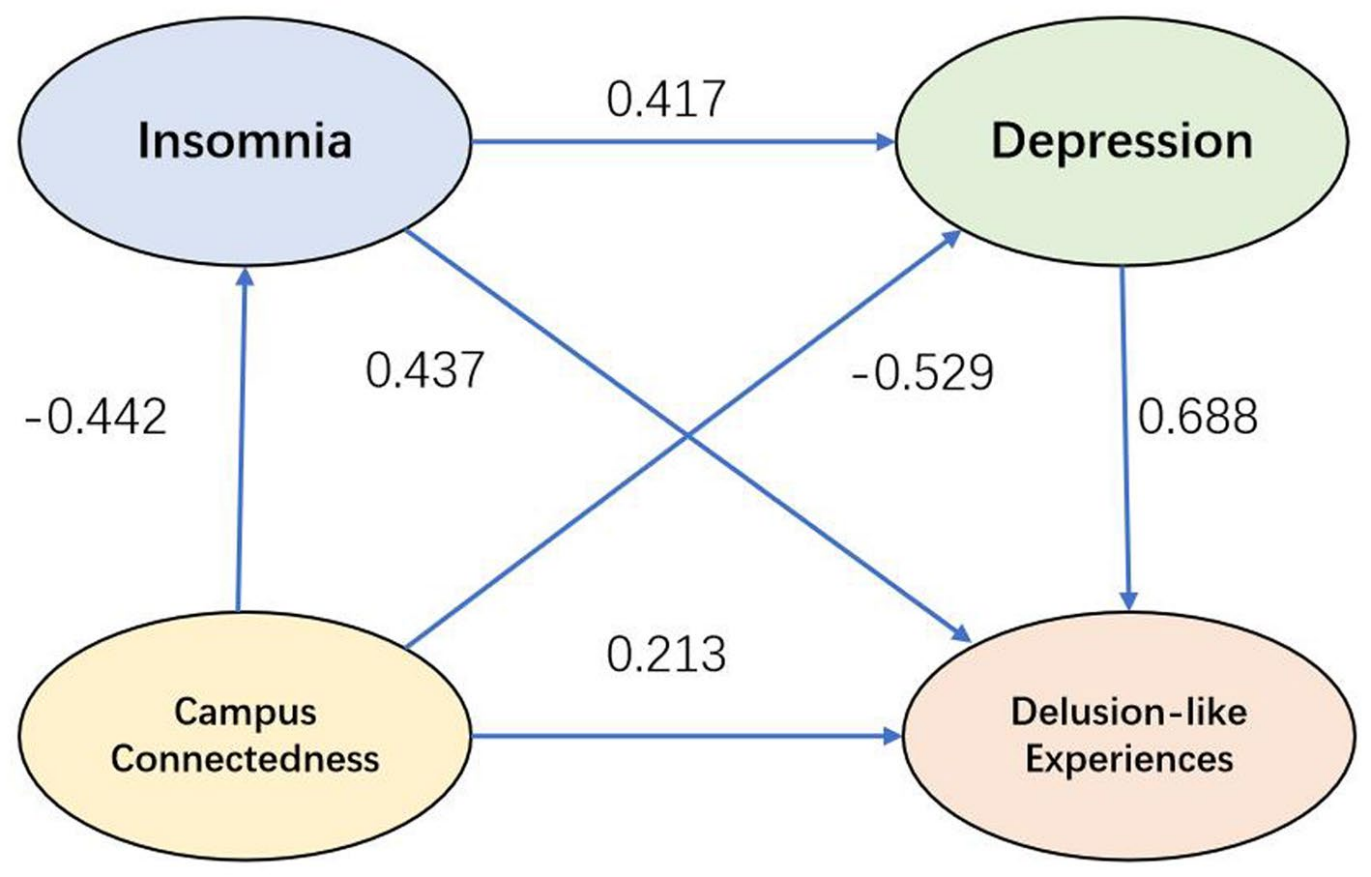
Schematic diagram of structural model path coefficients.

Figure 2 displays the regression paths of the structural model. Standardized regression coefficients among latent variables ranged from −0.529 to 0.688.

### 3.8 Mediation and suppression-like effects

Table 6 indicates that insomnia and depression exerted significant single and chained mediating effects between campus connectedness and delusion-like experiences (*p* < 0.001). Meanwhile, the direct effect of campus connectedness on delusion-like experiences was significant (*p* < 0.001), showing that insomnia and depression exerted an incomplete mediating effect in their relationship. Moreover, the direct effect shifted from negative to positive, indicating the presence of a suppression-like effect.

**Table 6:** Mediation and suppression-like effect analysis.

Mediation effect	Std.	Unstd.	Product of coefficients			BOOTSTRAP 5000 TIMES 95% CI	
			S. E.	Unstd./S. E.	*P*-value	Lower	Upper
CC— > INS— > DLEs	−0.193	−0.235	0.033	−7.169	<0.001	−0.304	−0.176
CC— > DEP— > DLEs	−0.364	−0.443	0.060	−7.343	<0.001	−0.579	−0.338
CC— > INS— > DEP— > DLEs	−0.127	−0.154	0.024	−6.301	<0.001	−0.212	−0.114
CC — > DLEs	0.213	0.260	0.059	4.388	<0.001	0.149	0.381

Std., standardized estimate; Unstd., unstandardized estimate; SE, standard error; Unstd./SE, ratio of unstandardized estimate to standard error; CC, campus connectedness; INS, insomnia; DEP, depression; DLEs, delusion-like experiences.

Overall, campus connectedness was negatively associated with delusion-like experiences, with insomnia and depression serving as both independent mediators and chain mediators. Furthermore, when insomnia and depression were included as partial chain mediators, the direct effect of campus connectedness on delusion-like experiences changed sign from negative to positive. These results were broadly consistent with the proposed hypotheses.

## 4 Discussion

Delusion-like experiences pose a significant threat to the mental health of college students. This study employed SEM to examine the negative association between campus connectedness and delusion-like experiences. Furthermore, the findings were consistent with significant indirect associations involving insomnia and depression, and a suppression-like effect was observed in the direct path between campus connectedness and delusion-like experiences.

In general, the results of this study were consistent with previous findings. Campus connectedness, referring to students’ sense of belonging to the university, perceived fit with others on campus, and feeling of social embeddedness within the campus community (Lee and Davis, 2000; Jouriles et al., 2020; Johnson and Plisco, 2025), was inversely associated with delusion-like experiences, consistent with prior research (Di Malta et al., 2022; Johnson and Plisco, 2025). Furthermore, the findings were consistent with significant indirect associations involving insomnia and depression. Specifically, lower campus connectedness was associated with higher levels of insomnia (Hsieh et al., 2019; Bao et al., 2022; Sun et al., 2025), and insomnia was in turn statistically associated with higher levels of delusion-like experiences (Goeder et al., 2021; Kammerer et al., 2021; Sheffield et al., 2021). Depression also showed a significant indirect association in the link between campus connectedness (Gaweda et al., 2013; Wilson et al., 2018; Raymond-Flesch et al., 2021) and delusion-like experiences (Khoury et al., 2023; Koposov et al., 2025). Additionally, Insomnia and depression were positively correlated (Chen et al., 2021; Hong et al., 2021; Jernelov et al., 2021). Taken together, these findings were consistent with a hypothesized chained indirect association linking campus connectedness, insomnia, depression, and delusion-like experiences. However, because the data were cross-sectional, this ordering should be interpreted as theory-informed rather than causally established.

A suppression-like pattern was observed in the association between campus connectedness and delusion-like experiences, as the residual direct path changed sign after insomnia and depression were included simultaneously. This pattern should be interpreted cautiously. Although the competing measurement models supported the empirical distinctiveness of the focal constructs and the auxiliary inner VIF results suggested low collinearity among predictors, the moderate-to-strong intercorrelations among insomnia, depression, and delusion-like experiences indicate that the sign reversal may still reflect the partitioning of shared variance among closely related mediators rather than a distinct substantive pathway. At the same time, prior research suggests that connectedness may, in some contexts, also be accompanied by psychological costs (Pesola et al., 2015; Sherman et al., 2020; Raniti et al., 2022), indicating that the relationship between connectedness and mental health may be more complex than a uniformly protective model would imply. Therefore, the present findings are better understood as pointing to a potentially complex and context-dependent association, which should be clarified in future studies using more fine-grained and longitudinal modeling approaches.

In the present study, sleep and emotional difficulties appeared to be important correlates of the association between campus connectedness and delusion-like experiences. Accordingly, the findings are consistent with the possibility that the association between campus connectedness and delusion-like experiences may partly operate through sleep- and mood-related difficulties among college students. At the same time, the observed suppression-like pattern suggests that this association may be more complex than a uniformly protective model would imply.

## 5 Limitations and future research

This study has several limitations. First, the cross-sectional design does not allow firm conclusions about temporal ordering or causality among the variables. To a large extent, the chained mediation model was theory-driven, and the proposed sequence linking campus connectedness, insomnia, depression, and delusion-like experiences cannot be established from the present data. Moreover, because some variables were moderately to strongly intercorrelated, the sign-reversed residual direct association in the full model should be interpreted cautiously, as it may reflect statistical suppression or overlapping variance rather than a stable substantive mechanism. In addition, some constructs were represented by dimension-based indicators derived from two-factor structures, which may have improved model fit while limiting the evaluation of item-level measurement properties. Furthermore, the primary model did not adjust for demographic covariates such as sex, age, grade, or higher education institution category. Although the model was theory-driven, residual confounding by these characteristics cannot be ruled out. Future research should use longitudinal designs, more suitable measures, finer-grained models, and theoretically justified covariate-adjusted model specifications to clarify temporal ordering and further examine the robustness of the suppression-like pattern. Second, the study relied primarily on self-report questionnaire data, which may be affected by recall bias, social desirability, response style, and limited insight into internal experiences. These factors may have introduced measurement error and may have attenuated or distorted the observed associations. Future studies should incorporate more diverse and potentially more reliable sources of data, such as clinician-rated assessments, behavioral indicators, sleep-monitoring data, or other multimethod approaches. Third, the sample was recruited through convenience sampling and snowball dissemination and was predominantly female, relatively young, largely composed of first-year students, and geographically concentrated. These characteristics may limit the representativeness of the sample and the generalizability of the findings. Future research should recruit more demographically diverse samples and, where feasible, adopt more rigorous sampling strategies, such as stratified or probability-based approaches.

## 6 Conclusion

This study examined the associations among college students’ campus connectedness, insomnia, depression, and delusion-like experiences. The findings were consistent with a hypothesized chained indirect association involving insomnia and depression. After both mediators were included, the residual direct association between campus connectedness and delusion-like experiences remained statistically significant but changed sign, suggesting a suppression-like pattern. Overall, the findings suggest that the association between campus connectedness and delusion-like experiences may be complex and context-dependent. However, the temporal ordering implied by the present model cannot be firmly established from the current data, and future research should use more fine-grained models to clarify the robustness and interpretation of the observed pattern.

## Data Availability

The raw data supporting the conclusions of this article will be made available by the authors, without undue reservation.
