# Supplementary material for: Paradoxical association between campus connectedness and delusion-like experiences among Chinese college students: a chained mediation
Source: Front Psychol. 2026 May 8;17:1769942. doi: 10.3389/fpsyg.2026.1769942 (PMC13194047; doi:10.3389/fpsyg.2026.1769942)
Supplement: Supplementary file 1 [file Table_S1.docx]

As an auxiliary check of multicollinearity, inner VIF values were examined in SmartPLS for the predictor constructs in the structural model. As shown in Table S1, all VIF values ranged from 1.000 to 1.939, which were well below both the conservative threshold of 3.0 and the commonly used cutoff of 5.0. These results suggest that collinearity among the predictors was low and unlikely to materially distort the structural path estimates.

**Table S1. Inner VIF values for predictor constructs in the structural model**

| **Structural paths** | VIF |
| --- | --- |
| CC -> DEP | 1.157 |
| CC -> DLEs | 1.597 |
| CC -> INS | 1.000 |
| DEP -> DLEs | 1.939 |
| INS -> DEP | 1.157 |
| INS -> DLEs | 1.426 |

**Note.** VIF, variance inflation factor; CC, campus connectedness; INS, insomnia; DEP, depression; DLEs, delusion-like experiences. Inner VIF values were obtained from an auxiliary SmartPLS analysis to assess collinearity among predictor constructs in the structural model.
