# Supplementary material for: Paradoxical association between campus connectedness and delusion-like experiences among Chinese college students: a chained mediation
Source: Front Psychol. 2026 May 8;17:1769942. doi: 10.3389/fpsyg.2026.1769942 (PMC13194047; doi:10.3389/fpsyg.2026.1769942)
Supplement: Supplementary file 2 [file Table_S2.docx]

As a supplementary robustness check, we re-estimated the mediation model with control variables included. The overall pattern of results remained substantively unchanged. As shown in Table S2, the indirect effects of campus connectedness on delusion-like experiences through insomnia (β = -0.189, 95% CI [-0.302, -0.173]), depression (β = -0.362, 95% CI [-0.578, -0.337]), and the sequential pathway of insomnia and depression (β = -0.123, 95% CI [-0.206, -0.109]) all remained statistically significant. In addition, the direct effect of campus connectedness on delusion-like experiences remained positive and significant (β = 0.207, 95% CI [0.139, 0.372]). These findings support the robustness of the main results.

**Table S2. Indirect and direct effects after inclusion of control variables**

| **Mediation effect** | **Std.** | **Unstd.** | **Product of Coefficients** | | | **BOOTSTRAP 5000 TIMES 95% CI** | | |  |
| --- | --- | --- | --- | --- | --- | --- | --- | --- | --- |
|  |  |  |  |  |  |  |  |  |  |
|  |  |  | **S.E.** | **Unstd./S.E.** | **P-Value** | **Lower** | **Upper** |  |  |
| **CC—>INS—>DLEs** | -0.189 | -0.231 | 0.033 | -7.058 | 0.000 | -0.302 | -0.173 |  |  |
| **CC—>DEP—>DLEs** | -0.362 | -0.442 | 0.061 | -7.283 | 0.000 | -0.578 | -0.337 |  |  |
| **CC—>INS—>DEP—>DLEs** | -0.123 | -0.150 | 0.024 | -6.192 | 0.000 | -0.206 | -0.109 |  |  |
| **CC** **—> DLEs** | 0.207 | 0.252 | 0.060 | 4.227 | 0.000 | 0.139 | 0.372 |  |  |

Std., standardized estimate; Unstd., unstandardized estimate; SE, standard error; Unstd./SE, ratio of unstandardized estimate to standard error; CC, campus connectedness; INS, insomnia; DEP, depression; DLEs, delusion-like experiences. Bootstrap confidence intervals were based on 5,000 resamples.
